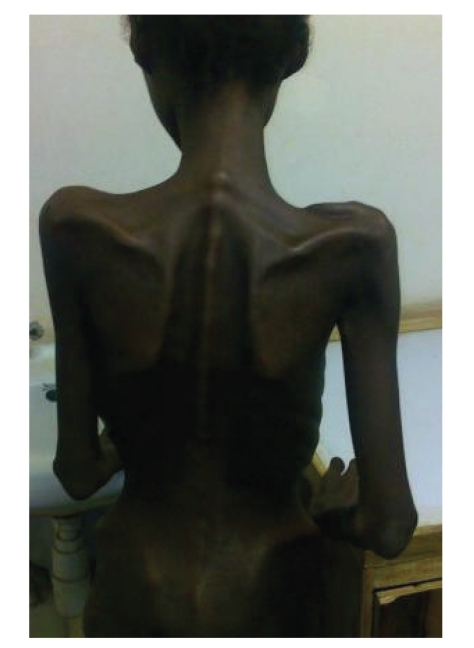# HIV Wasting Syndrome in a Nigerian Failing Antiretroviral Therapy: A Case Report and Review of the Literature

**DOI:** 10.1155/2010/192060

**Published:** 2010-12-27

**Authors:** Dimie Ogoina, Reginald O. Obiako, Haruna M. Muktar

**Affiliations:** ^1^Department of Medicine, Ahmadu Bello University Teaching Hospital (ABUTH), Zaria, Nigeria; ^2^Department of Haematology, Ahmadu Bello University Teaching Hospital (ABUTH), Zaria, Nigeria

## Abstract

The HIV wasting syndrome represented the face of HIV/AIDS before the advent of highly active antiretroviral therapy (HAART). Although the incidence of wasting has declined since the introduction of HAART, weight loss remains common in patients receiving HAART, especially in the setting of a failing HAART regimen. As we are not aware of any previous reports from Nigeria, we report a case of the classical wasting syndrome in a Nigerian female who had both virological and immunological HAART failure due to poor adherence. The influence of a failing HAART regimen, socioeconomic status, and other clinical variables in the wasting syndrome are discussed.

## 1. Introduction

HIV wasting syndrome has been defined by the Centre for Disease Control (CDC), USA, as involuntary weight loss greater than 10% of baseline weight associated with either chronic diarrhoea for at least 30 days or chronic weakness or documented fever for at least 30 days in the absence of a concurrent illness or condition other than HIV infection that could explain findings (e.g., tuberculosis, cryptosporidiosis, or other specific enteritis) [1] Although the wasting syndrome has declined since the introduction of highly active antiretroviral therapy (HAART) [2, 3], weight loss still remains a common cause of morbidity and mortality in HIV-infected patients receiving HAART [4, 5]. 

In Nigeria, weight loss and severe wasting often complicates HIV/AIDS [6–9], but we are not aware of any report specifically describing the risk factors, features, clinical course, and outcome of the wasting syndrome as defined by CDC, especially in patients receiving HAART. We describe a case of the classical wasting syndrome in an unemployed Nigerian widow and discuss the influence of a failing HAART regimen, socioeconomic status, and other clinical variables in the wasting syndrome.

## 2. Case History

A 32-year-old HIV-1-infected widow was admitted to our tertiary hospital with a 6-month history of progressive lethargy, anorexia, recurrent fever, vomiting, watery non-bloody diarrhoea, and progressive weight loss. She went from 50 kg to 21 kg in 5 months, a 42% weight loss. She was unemployed and had no source of regular income to care for herself or her 3 children. Hence, her meals before and during her illness were irregular and consisted mainly of carbohydrate diet. 

**Figure 1:**
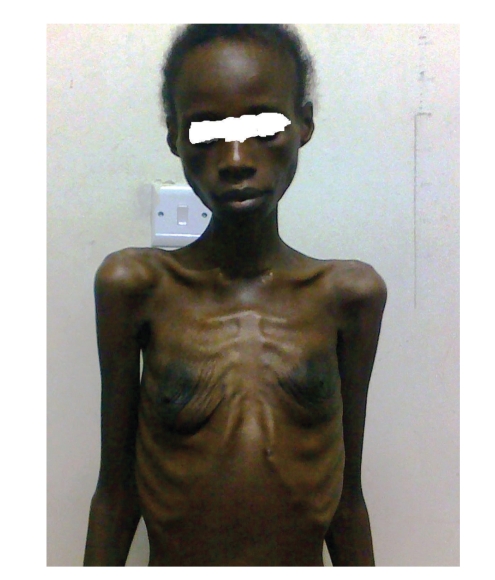


 On examination, she was conscious, prostrated, severely wasted with generalised wasting of muscle groups, loss of subcutaneous fat, and prominence of bones. There was no evidence of localised subcutaneous fat atrophy or fat redistribution. Her body mass index was 10.2 kg/m^2^, and the mid-upper arm circumference was 10 cm (Figures 1 and 2). She had hypoproteinemic hair and skin changes, dehydration, fever (37.8°C), pallor, and oropharyngeal candidiasis but no lymphadenopathy. Chest, cardiovascular, and abdominal examinations were normal. 

She was on HAART (Zidovudine, Lamivudine, Nevirapine) for 16 months but adherence was poor (<70%). On HAART, her viral reduced from 421,000 copies/ml to 41,000 copies/ml, but CD4+ T cell count dropped from 168 cells/ul to 34 cells/ul. Full blood count revealed microcytic hypochromic anaemia (packed cell volume of 24%) and leukopenia of 1.2 × 10^3^/ul. Three separate stool microscopy and culture, including modified Ziehl Neelsen stain for cryptosporidiosis and isosporiasis, were all negative for parasites and bacteria, and also negative for cells such as red blood cells and white blood cells. Apart from mild hypokalemia (serum potassium of 3.0 mmol/l) and low serum albumin of 22 mmol/l, other renal and liver function tests were normal. Chest X-ray and abdominopelvic ultrasound were normal. Hepatitis B and C serology and blood film for malaria parasites were also negative. 

She was rehydrated, and diarrhoea was controlled with an antimotility agent (tablets loperamide) after empiric treatment for infectious diarrhoea using tablets albendazole 400 mg daily for 3 days, intravenous ciprofloxacin 500 mg twice daily for one week, and tablets tinidazole 2 g start. Oral fluconazole 200 mg daily for oral thrush, prohylactic septrin 960 mg daily, multivitamins, and haematinics were also given. 

She had intensive nutritional rehabilitation with locally available high protein and high energy diet as well as fruits and vegetables. Specifically, “kwashiokor pap” or “kwashi pap”, a high protein local diet made up of a mixture of ground guinea corn, ground soya bean, and ground crayfish, ground dried fish, roasted ground groundnuts, and boiled water was given orally three to five times per day, along with variety of other local foods. 

Additional adherence counselling was given, and ART was switched to Emtricitabine/Tenofovir and Nevirapine on account of anaemia. 

All symptoms gradually resolved and she gradually regained her physical strength with improved physical activity. At the end of 6 weeks, her weight increased by about 8 kg and she was thereafter discharged to the social welfare, nutritional, and adherence counselling unit.

## 3. Discussion

 The pathophysiological mechanisms underlying the HIV wasting syndrome are related to three major factors including inadequate nutrient intake, nutrient malabsorption, and disturbances in metabolism (reviewed in references [10–12]). 

The burden of HIV infection impoverishes [13]; in sub-Saharan Africa, women and children are the most affected and they are more likely to suffer from neglect, discrimination, and abuse [14]. Poor socioeconomic status has been shown to be a strong determinant of weight loss in HAART experienced HIV-infected patients [4]. These patients were found not to be able to afford regular high-protein and high-calorie meals needed for weight gain and for restoration of lost body cell mass. In the reported case, poverty precluded adequate intake of nutritious meals before and during patient's illness and this problem was compounded during the illness by anorexia, oral sores, vomiting, and diarrhoea. Anorexia may also result from anxiety and depression, which are both common psychiatric complications of HIV infection [15]. Other recognised causes of inadequate nutrient intake include dysphagia and odynophagia, which may be due to infections of the oral cavity, posterior pharynx, or oesophagus. 

Both micronutrient and macronutrient malabsorption have been observed in the wasting syndrome, and nutrient malabsorption may occur with or without diarrhoea [10, 16]. Chronic diarrhoea leading to malabsorption may either be due to the HIV virus itself (i.e., AIDS enteropathy) or due to occult opportunistic infections (e.g., *Cytomegalovirus, Clostridium difficile, and Mycobacterium avium intracellular bacteria, *among others) [17]. In AIDS enteropathy, no specific pathogen can be isolated from the gut, and the chronic diarrhoea, motility disturbances, and mucosal atrophy accompanying this condition have been attributed to the direct effects of HIV, especially as viral proteins have been found in the gut mucosa [18]. AIDS enteropathy is a diagnosis of exclusion; however, complete exclusion of all opportunistic causes of diarrhoea in HIV-infected patients is a challenging task sometimes requiring invasive techniques [17, 19]. Consequently, the WHO recommends the use of empiric antimicrobials and constipating drugs such as loperamide, for treatment of chronic HIV-related diarrhoea in resource poor settings [20]. 

By leading to malabsorption and/or changes in pharmacokinetics of the ART medications, chronic diarrhoea may also contribute to HAART treatment failure. In a clinical study by Brantley et al. [21], AIDS-related diarrhoea and weight loss were associated with both subtherapeutic plasma levels of antiretroviral medications and protozoan pathogens in stool. Hence, in patients receiving HAART, prompt and effective treatment of chronic diarrhoea is essential to prevent both weight loss and HAART treatment failure. 

Various metabolic abnormalities leading to weight loss have been described in HIV-infected patients, and these abnormalities have been attributed to several etiologies including the HIV virus itself, concomitant opportunistic infections, cytokine dysregulation, and hormonal imbalances accompanying HIV infection, as well as ART medications [10, 11]. 

A failing HAART regimen leads to persistent viral replication and progressive immunosuppression as reflected in the CD4 cell count. Correlations have been established between high HIV viral load, low CD4 cell counts, and weight loss [4, 22, 23]. In HAART experienced patients with suppressed plasma viral load, weight loss has been attributed to the persistence of HIV in peripheral blood monocytes and macrophages [24]. The persistence of HIV leads to excessive cytokine activation and dysregulation, and this in turn triggers various metabolic abnormalities that lead to weight loss such as increase in resting energy expenditure, proteolysis, and hypercatabolism. Cytokines may also inhibit anabolism by causing growth hormone resistance and by reducing hepatic production of insulin-like growth factor-1, a messenger of growth hormone [25]. The increase in resting energy expenditure and cytokine dysregulation in HIV infection may be intensified by concomitant opportunistic infections [10]. Various cytokines, such tumour necrosis factor *α*, interleukins-1 (IL-1), IL-6, and interferon gamma, have been implicated in these metabolic perturbations [11]. 

By interfering with lipid metabolism in muscle, elevated levels of proinflammatory cytokines such as TNF-*α* also lead to muscle weakness and muscle atrophy [26]; both are characteristics features of the wasting syndrome. 

The use of HAART has also been independently associated with an increase in resting energy expenditure, and this has been suggested as one of the factors perpetuating weight loss in the HAART era [4, 10]. Although sometimes challenging to distinguish, even with appropriate body composition measurements, HAART-induced weight loss often leads to loss of fat loss and/or fat redistribution (Lipodystrophy) with little or no loss of lean body mass [10]. Conversely, the wasting syndrome is characterised by a complex interplay of lean body and fat loss depending on baseline body weight and other factors such as gender [10, 27]. With progressive HIV infection, women lose a higher amount of body fat relative lean body mass while men lose more LBM than fat [27]. These gender differences in weight loss have been attributed greater premorbid fat stores in women than men, as well as due biological and hormonal factors [27]. 

 Hypogonadism, represented by low levels androgen such as testosterone, may accompany HIV infection in men [28], and it may be as a result of the suppressive effects of cytokines on testicular steroidogenesis, as well as due to functional disorder of the hypothalamus and/or primary testicular failure [29]. Androgen deficiency inhibits protein synthesis and this may favour a greater loss of muscle mass relative to fat mass in men. In women, testosterone levels are normally low and although androgen deficiency has been reported in some women with weight loss, their contribution to weight loss in women is less understood [30].

Intensive nutritional rehabilitation to prevent or reverse weight loss remains the cornerstone of management of the wasting syndrome [10, 12]. The aims are to improve appetite and nutrient absorption by addressing all immediate causes of anorexia and malabsorption, such as oral sores and diarrhoea, to improve intake of adequate calories made up of high-protein and low-fat meals in addition to micronutrients supplementation, and to correct psychosocial issues that affect nutrient intake such as poverty and depression, by providing social and psychological support. In view of the principal role of HIV in the pathogenesis of the wasting syndrome, effective HAART aimed at reducing viral load to undetectable levels and sustaining immune restoration as reflected in improving CD4 cell count is indispensable [12]. 

In combination with adequate caloric intake, fitness training by progressive resistance exercises (e.g., lift of light weights and body building exercises) increases muscle function and strength, as well as lean body mass and weight [31]. Conversely, aerobic exercises (e.g., walking, jogging, and running) may result in little or no increase in body mass or weight. 

Pharmacological treatments are usually reserved for patients who fail nutritional therapy. Appetite stimulants (e.g., Megestrol acetate), recombinant human growth hormone (Serostim), and androgenic steroids (e.g., testosterone) in men with hypogonadism have all been approved for the treatment of the wasting syndrome [12]. Cytokine modulators (such as thalidomide) have been investigated for treatment of wasting syndrome, but their success rates have been variable [11, 12, 32]. Thus, they are not yet approved for the management of the wasting syndrome, until convincing evidence of their efficacy is established in future studies.

## 4. Conclusion

HIV wasting syndrome is a disorder characterised by multiple pathophysiological mechanisms, most mediated by the HIV virus itself and driven by nutritional abnormalities. HAART treatment failure is an emerging global challenge, especially in developing countries where HIV infection is still endemic and the use of HAART is being scaled up [33]. A failing HAART regimen leads to HIV persistence and acting together with psychosocial issues such as poverty, it sets the stage for the re-emergence AIDS defining illness such as the wasting syndrome.

To prevent or reverse the resurgence of AIDS defining illness such as the wasting syndrome in this HAART era, ensuring effective uninterrupted HAART, socioeconomic empowerment of HIV-infected patients, and prevention of opportunistic infections are priorities for developing countries such as Nigeria.

##  Author's Contributions

We declare that this work was done by the authors and all liabilities pertaining to claims relating to the content of this article will be borne by the authors. 

The first author conceived the report; all authors were involved in patient management, manuscript preparation, and review, and they approved the final version for publication.

##  Conflict of Interests

No conflict of interests associated with this work.

##  Ethics

Consent was obtained from the patient for clinical photos.

## Figures and Tables

**Figure 2:**